# Where do we need to improve resuscitation? Spatial analysis of out-of-hospital cardiac arrest incidence and mortality

**DOI:** 10.1186/s13049-023-01131-8

**Published:** 2023-10-26

**Authors:** Robin Buter, Hans van Schuppen, Hendrik Koffijberg, Erwin W. Hans, Remy Stieglis, Derya Demirtas

**Affiliations:** 1 Center for Healthcare Operations Improvement and Research, University of Twente, Drienerlolaan 5, Enschede, 7500 AE the Netherlands; 2 Industrial Engineering and Business Information Systems, University of Twente, Drienerlolaan 5, Enschede, 7500 AE the Netherlands; 3 Department of Anesthesiology, Amsterdam UMC Location University of Amsterdam, Meibergdreef 9, Amsterdam, 1105 AZ the Netherlands; 4 Health Technology & Services Research, University of Twente, Drienerlolaan 5, Enschede, 7500 AE the Netherlands

## Introduction

Improving survival from out-of-hospital cardiac arrest (OHCA) is an important public health challenge. Overall survival is low and varies widely per country [1, 2]. Over the years, actions to improve survival have targeted different aspects in the chain of survival [3]. These actions often focus on logistics to provide rapid basic life support (BLS) and defibrillation [4]. For example, volunteer responder systems (VRS) have been implemented in several countries, dispatching trained volunteer responders to start cardiopulmonary resuscitation (CPR) with the use of automated external defibrillators (AED) [5, 6]. These systems have been shown to increase CPR before ambulance arrival, decrease time to first defibrillation, and increase survival rates for OHCA patients [7, 8].

A good understanding of where and when OHCAs occur is important for effectively directing public health resources aimed at improving survival. To fasten defibrillation by bystanders or volunteer responders, public access defibrillators need to be positioned close to the OHCA and need to be accessible at all times [9–13]. Therefore, insight into the spatial and spatiotemporal incidence of OHCA is crucial to place AEDs effectively, but is often lacking. Previous studies have shown that a probability distribution of spatial incidence of OHCA can be used as input to analytical models to find the best locations for AEDs [14–16]. In addition, identifying geographical areas that have disproportionally many non-survivors compared to survivors (and vice versa) allows for further specific targeting interventions to increase survival, like local public awareness campaigns.

Previous spatial analysis methods have provided significant, but limited insight. Recent studies that analysed spatial or spatiotemporal OHCA risk used spatial analysis methods [17–19] or Bayesian methods [20–25]. All these methods require aggregating data in spatial cells or administrative areas, which means that results and granularity are influenced by that choice. Furthermore, several studies investigated how the spatial distribution developed over the years [19–22]. It is known that temporal (in)accessibility of AEDs is an important aspect in effective defibrillation by bystanders [10]. Additionally, (spatial) availability of volunteer responders may depend on the time of day, as people go to work and change locations throughout the day. Therefore, we are interested in how spatial incidence develops throughout the day, instead of over the years.

The objective of this study was to propose a methodology (1) to analyse how incidence of OHCA is distributed throughout a study region, (2) to analyse how incidence changes over time of day, and (3) to identify which areas have significantly more survivors or non-survivors and explore to what extend basic case characteristics explain any difference found. We applied this method to a case study of OHCA in Amsterdam, the Netherlands, to show how the method works in practice.

## Methods

### Setting

This study screened all resuscitation attempts for OHCA with presumed cardiac cause in the municipality of Amsterdam during the period 2006–2016. When an emergency call was made to the dispatch centre because of an OHCA, both first responders (police and fire fighters) and two ambulances were dispatched to the scene. Police and fire fighters were trained in BLS and vehicles were equipped with AEDs. During the study period supraglottic airway devices were increasingly used for advanced airway management by EMS during prehospital advanced life support, instead of tracheal intubation. No other major changes in prehospital resuscitation practice were implemented during the study period. In 2016, the municipality of Amsterdam had a population of 833,624 inhabitants [26].

### Data collection

This study used data collected by AmsteRdam Resuscitation STudies (ARREST). ARREST is an ongoing prospective registry of all-cause OHCA in the Dutch province of North-Holland since 2005. The routine data collection, which includes data on the GPS location of the OHCA and data from dispatch centre, AEDs and emergency medical service (EMS) (including manual defibrillator data), and informed consent procedure is described extensively elsewhere [27, 28]. The ARREST data collection is approved by the Institutional Review Board of the Academic Medical Center of Amsterdam.

Missing GPS coordinates were derived from the OHCA addresses using Google Maps Platform API. Subsequently, GPS coordinates were converted to X and Y coordinates on the projected coordinate system EPSG:28,992. Municipal boundaries in 2016 were obtained from geographic information system files published by Kadaster and Central Bureau for Statistics (CBS) [29]. A case was considered to be in a residential area when the OHCA was located in or around a residential place (including nursing homes). A shockable initial rhythm was defined as ventricular fibrillation or ventricular tachycardia, confirmed by an AED or a manual defibrillator of the EMS. Defibrillator connection time was defined as the time between initiation of the emergency call and the first connection of either an AED or a manual defibrillator.

### Statistical methods

We used a statistical smoothing technique called kernel density estimation (KDE) to analyse and visualise the incidence of OHCA and compare mortality across the study region. This subsection provides a brief introduction into the methods, with additional details available in Supplementary Material 1.

KDE puts a smoothing curve (kernel function) on top of each data point to estimate the underlying probability density function of the data. A commonly used kernel function is the normal kernel (Gaussian), which is centered at each data point and scaled by a bandwidth parameter that determines the degree of smoothing. The probability density function of the data can then be estimated by calculating the sum of the contributions of all kernels at any point in the study region. This produces a smoothed-out estimate of the probability density function of the data, where areas with high density (i.e. many data points) have a large bump in the estimate, and areas with low density (i.e. few data points) have a smaller bump. KDE is well explained in Chacón and Duong’s book [30].

*(1) Spatial distribution of OHCA incidence*.

For the spatial analysis, we used a two-dimensional KDE model with normal kernels. We defined high incidence areas as the smallest regions encompassing 20% of the total probability density, and these areas were indicated by contour lines in the visualisation. The spatial analysis only requires coordinates of historic OHCA cases, which could be obtained from dispatch centres, EMS, VRS, or a cardiac arrest registry (like in this study, ARREST).

*(2) Spatiotemporal distribution of OHCA incidence*.

The spatiotemporal model extends the spatial model by incorporating a temporal dimension [31]. The temporal dimension was defined as time of day (00:00–24:00), which is circular like a clock (e.g. 21:00 and 01:00 are both be equally close to 23:00). To model this correctly, von Mises kernel was chosen to model the temporal dimension [32], which is an analogue to a normal distribution on a circle. The spatiotemporal analysis requires coordinates and timestamp of OHCA, which could be obtained from the same source as the data for the spatial analysis.

*(3) Spatial relative risk of mortality*

Spatial relative risk can be used to compare the spatial distribution of non-survivors with the spatial distribution of survivors. We calculated the natural logarithm of the kernel density ratio, i.e. the ratio of estimated probability density of non-survivors to survivors [33]. Areas of statistically significant high or low risk of mortality were indicated by contour lines at significance level of 5%, calculated using R package *sparr* [34]. Identifying the high or low risk areas requires coordinates and binary survival outcome of OHCA.

Additionally, we conducted logistic regression analysis to examine the impact of basic case characteristics (age, sex, public location, witnessed, and shockable rhythm) on the survival differences between high and low-risk areas. Odd ratios and 95% confidence interval (95% CI) were calculated. Risk areas (low, neutral, high) were treated as categorical variables, both with and without adjustment for the aforementioned basic case characteristics. The reference group for high and low-risk areas were the OHCAs in the neutral-risk area.

## Results

**Fig. 1:**
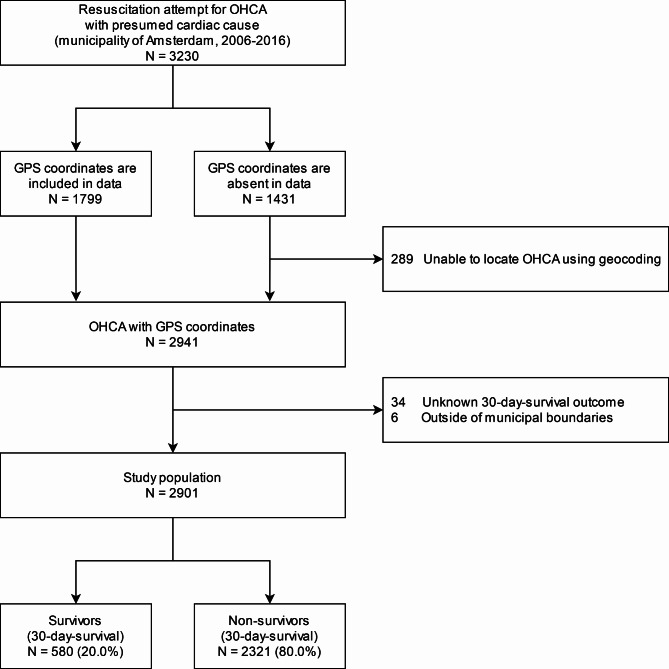
Flowchart of included and excluded out-of-hospital cardiac arrests, municipality of Amsterdam, 2006–2016. OHCA: out-of-hospital cardiac arrest, GPS: Global Positioning System

We applied our methods to a case study of Amsterdam. In total 3230 OHCAs with presumed cardiac cause were obtained from the ARREST database. After excluding cases with missing coordinates or survival outcome, 2901 OHCAs remained as the study population, of which 20% survived after 30 days (Fig. 1). Results based on just the five most recent years of data (i.e. 2012–2016 instead of 2006–2016) showed a similar picture and can be found in Supplementary Material 2.

### Spatial distribution

**Fig. 2:**
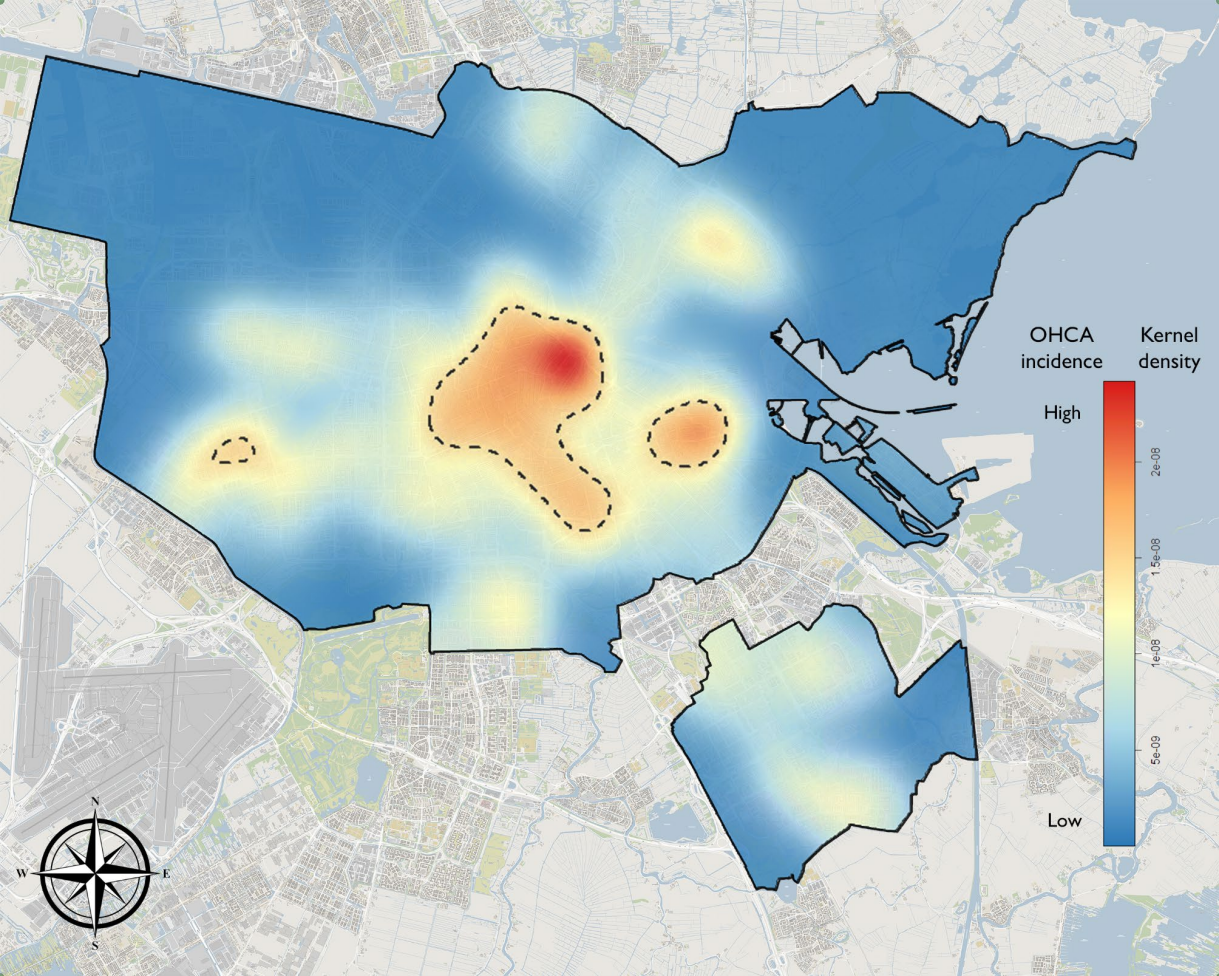
Visualisation of out-of-hospital cardiac arrest incidence in Amsterdam. Spatial kernel density estimation of out-of-hospital cardiac arrest incidence in Amsterdam. The contour lines indicate high incidence areas, defined as the smallest total area that encompasses 20% of the total probability density. (Municipal boundaries: © Kadaster / Central Bureau for Statistics, 2018, CC BY-SA. Background: © OpenStreetMap contributors, CC BY-SA.)

Figure 2 shows the spatial distribution of OHCA incidence. The high incidence areas are indicated by contour lines and occurred in the Amsterdam city centre and east and west of the city centre. In the high incidence areas historic OHCA incidence was 5.6 OHCA/km^2^/year, while outside those areas incidence was 1.07 OHCA/km^2^/year.

In addition to the high incidence areas, there were medium cluster of incidence (yellow/orange colour) northeast and south of the city centre, and in the southeast part of Amsterdam.

### Spatiotemporal distribution

**Fig. 3:**
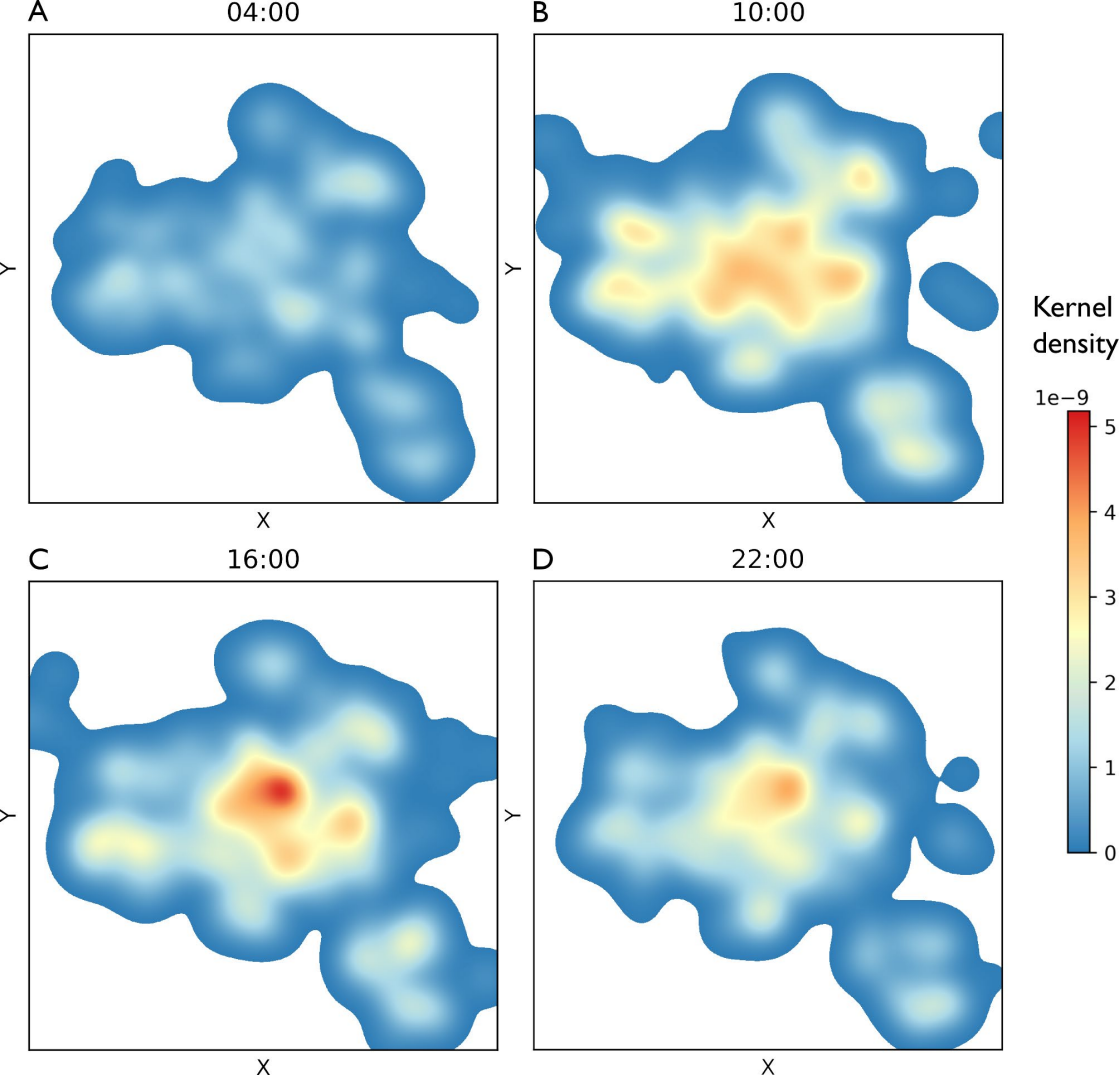
Visualisation of out-of-hospital cardiac arrest incidence in Amsterdam throughout the day. Spatiotemporal kernel density estimation of out-of-hospital cardiac arrest incidence in Amsterdam. Panels A, B, C, & D show the spatial distribution at specific time points 04:00, 10:00, 16:00, and 22:00 h, respectively

Figure 3 shows slices of the spatiotemporal distribution at 04:00, 10:00, 16:00, and 22:00, indicating how the spatial distribution changes over time of day. In the morning, at 04:00 and 10:00, incidences were more uniformly spread over the city compared to the afternoon (16:00) and evening (22:00). The overall incidence was relatively lower during the night and in the morning, but was not insignificant. The city centre became a hotspot in the afternoon. Supplementary Material 3 shows an animation of the spatial distribution over 24 h.

### Spatial relative risk of mortality

**Fig. 4:**
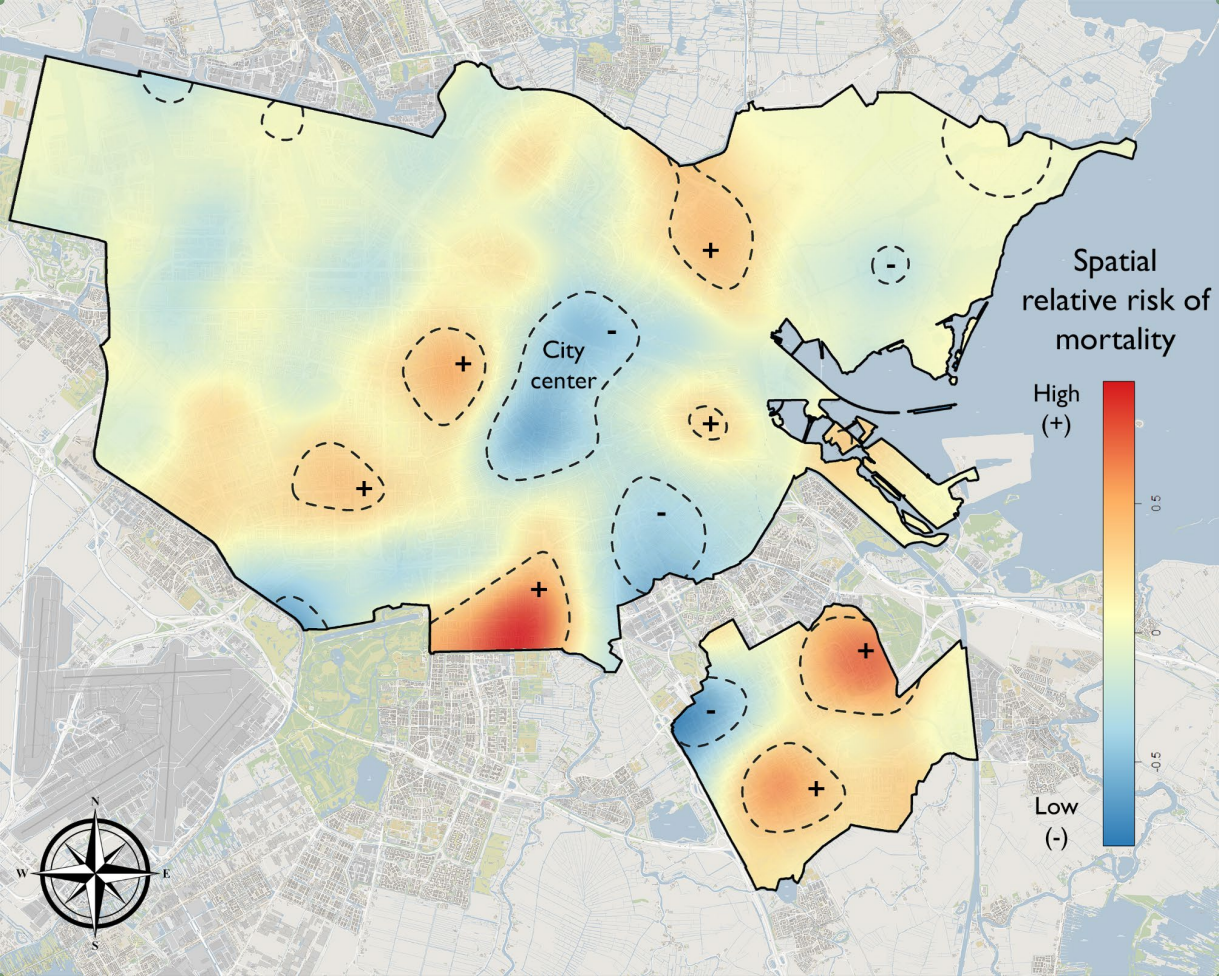
Spatial relative risk estimate of out-of-hospital cardiac arrest mortality in Amsterdam. Contour lines indicate areas of statistically significant high (+) or low (-) relative risk of mortality. Contour lines were obtained by a two-sided statistical test at significance level 5%. Note that the + and - symbols refer to the (log) spatial relative risk scores being either positive or negative values, and not to a desired outcome. (Municipal boundaries: © Kadaster / Central Bureau for Statistics, 2018, CC BY-SA. Background: © OpenStreetMap contributors, CC BY-SA.)

We calculated the spatial relative risk estimate for mortality after OHCA in Amsterdam (Fig. 4). The areas of interest are indicated by contour lines obtained by a two-sided statistical test (α = 5%), with the (+) and (-) symbols referring to the sign of the estimate. The areas with low relative risk of mortality, indicated by (-) in Fig. 4, are referred to as high-survival areas, and the areas with high relative risk of mortality, indicated by (+), are referred to as low-survival areas. Areas which were classified as neither high- nor low-survival are referred to as neutral-survival areas.

The high-survival areas were mainly located in the city centre and south-east of the city centre. Low-survival areas could be found surrounding the city centre. A particularly intense low-survival area was located south of the city centre, spatially aligned with the residential area of a specific neighbourhood. In the disjoint south-east part of the municipality, two low-survival areas could be seen, in addition to one high-survival area in which primarily businesses, stores, and a large football stadium are located.

A total of 432 (14.9%) and 562 (19.4%) cases occurred in the identified high- and low-survival areas, respectively (Table 1). When comparing high- and low-survival areas, significant differences in 30-day survival were found in the whole study population (35.6% vs. 9.1%) and in the subset of the Utstein comparator group (witnessed arrests with shockable rhythm, 59.5% vs. 25.9%). Notably, there were differences in shockable initial rhythm, CPR before EMS and public location between the high- and low-survival areas. The difference in the AED connection rate was not statistically significant. However, for cases in which an AED was connected before EMS, it concerned a local AED in 32.7% of the cases in the high-survival areas, compared to only 5.1% of the cases in the low-survival areas.

**Table 1:** Characteristics of out-of-hospital cardiac arrests in the high-, neutral-, and low-survival areas

Variables	High-survival	Neutral-survival	Low-survival	Total	*P*
**n**	432	1907	562	2901	
**Median age (IQR)**	63 (53–72)	66 (55–77)	70 (60–81)	67 (56–77)	*< 0.001*
**Male**	81.3% (of 432)	67.5% (of 1907)	64.1% (of 562)	68.9% (of 2901)	*< 0.001*
**Public**	58.8% (of 432)	24.0% (of 1906)	15.8% (of 562)	27.6% (of 2900)	*< 0.001*
**Witnessed**	79.2% (of 423 )	72.5% (of 1872)	70.1% (of 556)	73.0% (of 2851)	*0.002*
**CPR before EMS**	78.3% (of 414)	68.5% (of 1843)	66.4% (of 548)	69.6% (of 2805)	*< 0.001*
**AED before EMS**	52.3% (of 432)	47.1% (of 1906)	48.4% (of 562)	48.1% (of 2900)	*0.25*
**Local AED**	32.7% (of 206)	13.3% (of 897)	5.1% (of 272)	14.8% (of 1395)	*< 0.001*
**Shockable initial rhythm**	55.3% (of 409 )	38.5% (of 1828)	32.0% (of 544)	39.7% (of 2781)	*< 0.001*
**30-day-survival**	35.6% (of 432)	19.7% (of 1907)	9.1% (of 562)	20.0% (of 2901)	*< 0.001*
**30-day-survival (Utstein)**	59.5% (of 205)	45.8% (of 609)	25.9% (of 147)	45.7% (of 961)	*< 0.001*

Unknown or missing values were excluded for each variable separately and the numbers in parenthesis indicate the number of data points for each variable. CPR and AED rates reflect BLS and AED use before EMS arrival. Local AED refers to an on-site AED (instead of AEDs brought by first responders) and the corresponding value is defined only for the subgroup of cases where an AED is connected. 30-day survival is also given for the Utstein comparator group, i.e. witnessed arrests with shockable rhythm. All P-values except for age were obtained by a two proportion z-test comparing high and low-survival. P-value for age was obtained by a Kruskal-Wallis test. CPR: cardiopulmonary resuscitation, AED: automatic external defibrillator

The median time to defibrillator connection in high-survival areas was 1:37 min shorter compared to low-survival areas (Table 2). The difference between the median time to shock was almost 2 min in favour of the high-survival areas.

**Table 2:** Defibrillator connection time and time to first shock of out-of-hospital cardiac arrests in the high-, neutral-, and low-survival areas

Variables		High-survival	Neutral-survival	Low-survival	*P*
**Defibrillator connection time (min)**	**n**	370	1700	504	
	**Mean**	08:21	09:40	10:06	< 0.001
	**Median (IQR)**	07:41 (05:54 − 10:06)	09:02 (07:00–11:45)	09:17 (07:10–11:53)	
**Time to first shock (min)**	**n**	203	652	156	
	**Mean**	08:25	09:49	10:28	< 0.001
	**Median (IQR)**	07:49 (05:56 − 10:13)	09:09 (07:04–11:41)	09:43 (07:12–12:24)	

Defibrillator connection time is retrieved from either a connected automatic external defibrillator or from the manual defibrillator of the Emergency Medical Service. Values exceeding 30 min were excluded from analysis in this table. P-values were obtained by a Kruskal-Wallis test.

To (partially) explain the differences in survival between the identified areas, we applied logistic regression on 30-day-survival. Cases with any missing value for the variables were excluded (n = 169). Without controlling for case characteristics, the odds of 30-day-survival in the high-survival area was 2.40 (95% CI 1.90–3.03, *P* < 0.001) times that of the survival in the neutral-survival area, whereas OR for the low-survival area is 0.36 (95% CI 0.26–0.50, *P* < 0.001). Controlling for case characteristics, the adjusted OR for the high-survival area becomes 1.44 (95% CI 1.08–1.92, *P* = 0.01) and adjusted OR for the low-survival area becomes 0.41 (95% CI 0.28–0.58, *P* < 0.001).

## Discussion

### Key findings

This study used KDE-based models to investigate the spatial distribution of OHCA incidence, the spatiotemporal distribution of OHCA incidence over time of day, and the spatial relative risk of survival. The methods are generalizable to any city with OHCA data, but were applied to a case study of Amsterdam as an example.

From the spatial model we identified several high incidence clusters, especially the city centre. The spatiotemporal model showed how incidence is distributed more evenly over the city during night and morning, whereas from afternoon onwards the city centre becomes the main hotspot. Using spatial relative risk, we were able to cluster OHCAs and identify multiple areas with significant differences in 30-day survival, merely based on their geographical location and survival outcome. For the Utstein comparator subgroup, we observed a significant difference as well. After adjusting for basic case characteristics (as defined in Sect. 2.3), the adjusted OR of the survival areas were still statistically significant.

### Spatial relative risk

Possible explanations for the spatial differences in survival can be divided into two categories: case characteristics are different (i.e. age, witnessed arrest, shockable rhythm, etc.) and/or treatment (CPR and AED use before EMS arrival, EMS treatment, hospital treatment, etc.) is different. Regarding case characteristics; numerous studies have linked age [35] and socioeconomic status [36, 37] with OHCA incidence and mortality. Our logistic regression analysis revealed that case characteristics could help explain difference in mortality, but only to a certain extend. Possibly, other differences in case characteristics like socio-economic status, can further explain spatial difference in mortality.

Differences in the performance of the chain of survival could help explain remaining differences in survival. We found a lower bystander defibrillation rate and local AED use in the low-survival areas (Table 1). Despite that AED connection rate before EMS arrival was not significantly different in the high- and low-survival areas, the defibrillator connection time and time to first shock was approximately two minutes shorter in the high-survival areas compared to the low- and neutral-survival areas (Table 2).

Repeating the spatial relative risk analysis using only the most recent 5 years of data (2012–2016) yielded results akin to those obtained using the complete 11-year dataset (see supplemental materials). The survival disparity remained approximately the same, as did the proportion of data points in the low and high survival areas. This result is consistent with the observation that there were only small fluctuations in yearly survival rates in Amsterdam,

### Implications for the chain of survival

The spatial analysis methods based on KDE can provide valuable information on areas that need improvement in resuscitation attempts. We observed that OHCA cases in low-survival areas were mainly residential with an AED connection rate similar to the other identified areas, but with longer connection and shock times. Efforts should be made to further increase CPR before EMS arrival and achieve faster defibrillation. It is notable that during the study period, unlike a major part of the Netherlands, a VRS was not yet used in Amsterdam, which could improve time-to-defibrillation, particularly in the low-survival areas.

The low-survival areas may be targeted with specific interventions to improve health outcomes. Current AED coverage, including temporal accessibility (and possibly signage [38]), should be evaluated first. A simple approach could be to evaluate AED density per km^2^ [11], and to consider placing more AEDs in the areas with low AED density, though placing AEDs at the right location would remain as a challenge. To that purpose, the spatial and spatiotemporal models can serve as input for mathematical models for strategic AED placement. These models also give information on the performance of existing AEDs and the marginal benefit of positioning additional AEDs. Similarly, the models presented in this paper can serve as input to ambulance location and allocation models which aim to place ambulances in high risk areas to decrease response time.

In addition to deploying AEDs and implementing a VRS, the volunteer responder density should be evaluated. A recent study recommended a density of > 10 available volunteers per km^2^ in residential areas [11]. Allocating funds to organize local awareness campaigns or to reimburse resuscitation courses may be more impactful than purchasing additional AEDs. After all, AEDs must be retrieved and connected by trained volunteer responders.

Furthermore, when deciding which areas to target, it is worth considering different levels of significance of the statistical test depending on available budget, cost, and scalability of the intervention. Increasing the significance level α of the statistical test increases the surface area of the high- and low-survival areas. If resources are limited, one could instead first try lower α to target (smaller) areas with the highest risk of mortality.

Lastly, the models presented in this paper are generalizable to any city or region, but the analyses require access to an OHCA registry with at least a few years of OHCA data with location and time information, which is not commonly available. The proposed spatial relative risk analysis also requires survival outcome and preferably case characteristics. Nevertheless, nowadays, locations and timestamps may be more easily obtained from smartphone apps that dispatch community volunteers (e.g. HartslagNu, GoodSAM, PulsePoint).

### KDE compared to other spatial analysis methods

Previous studies that analysed spatial or spatiotemporal OHCA risk used models that aggregate data into spatial cells [17–25, 39]. Spatial analysis methods, such as the Getis-Ord Gi* statistic, were used to identify high-risk census tracts [17–19]. Another common approach was to use a Bayesian model with parameters for spatial and temporal heterogeneity, space-time interactions, and demographic covariates [20–24]. It is clear that the way in which spatial cells are defined impacts or limits the analyses and results.

A major advantage of KDE is that it is a continuous estimate and does not require delimitation of the study region into spatial cells. Therefore, the boundaries of the said spatial cells or administrative areas do not restrict the analysis or influence the results. Additionally, discrete models assume uniform incidence throughout spatial cells and therefore may result in abrupt transitions between and around the borders of these cells. The scope of possible analyses using KDE is flexible, as it can be done on a country, province, city, or even district level, without requiring population data on that scope.

### Limitations

Survival data for OHCAs is inherently imbalanced. In our case, 30-day survival of 20.0% is relatively high compared to other countries. If survival is extremely low, like < 5%, then the spatial relative risk method may not be accurate, since there are too few data points of patients who survived. However, if survival is that low, the spatial distribution alone already provides sufficient insights.

A spatial plot (i.e. Figure 2) provides additional context for the spatial relative risk analysis, giving additional insight into the overall incidence in that vicinity and thus the importance of that finding. The spatial relative risk analysis does not consider the overall incidence. This means that an area can be identified as low (or high) survival but may locally have relatively few data points. For example, the two north-west clusters with contour lines near the boundary in Fig. 4 represent only a few isolated points, thus indicating low importance. Therefore, results from the spatial relative risk model should be interpreted with the spatial distribution in mind.

### Future directions

First, a study further investigating the cause of severe disparity in survival outcomes may prove useful. After adjusting for case characteristics, the OR of the survival areas were still far from 1 and statistically significant, indicating that we cannot fully explain survival (differences) with just the case characteristics. A more comprehensive approach taking for example comorbidities, socioeconomic status, local stakeholders and local public health experts’ knowledge into account may be necessary to understand the underlying reasons for differences in survival. Second, analysing and modelling spatiotemporal volunteer responder availability would be valuable to further improve the chain of survival. Third, our results support the development of a dashboard to monitor regional OHCA incidence and care and to identify where additional effort can be made to improve public health. Lastly, the Dutch VRS was implemented in Amsterdam in 2019, so prospective data can help direct further improvements and assess impact on survival.

## Conclusions

KDE is useful to identify areas of interest regarding spatial OHCA incidence, spatiotemporal OHCA incidence, and spatial relative risk of OHCA mortality. Further causal analysis and engagement with local stakeholders and public health experts are the next steps in understanding disparities in survival between geographical areas. Results motivate where additional public health efforts to improve resuscitation attempts should be focused.

### Electronic supplementary material

Below is the link to the electronic supplementary material.

Supplementary Material 1

Supplementary Material 2

Supplementary Material 3

## Data Availability

The datasets generated during and/or analysed during the current study are not publicly available due to privacy regulations. Any questions or requests regarding the data should be directed to ARREST.
