# Supplementary material for: Where do we need to improve resuscitation? Spatial analysis of out-of-hospital cardiac arrest incidence and mortality
Source: Scand J Trauma Resusc Emerg Med. 2023 Oct 26;31:63. doi: 10.1186/s13049-023-01131-8 (PMC10605336; doi:10.1186/s13049-023-01131-8)
Supplement: Supplementary file 1 — Supplementary Material 1 [file 13049_2023_1131_MOESM1_ESM.docx]

This document gives additional information and details on the methods and modeling choices (Methods), and the selected bandwidth values (Result).

**Methods**

*Kernel density estimation*

The research questions of this study were answered using models based on KDE, which is a nonparametric statistical smoothing approach to estimate an unknown continuous probability density function. It does so by placing a kernel function on top of each data point to smooth out the contribution by each point and then by aggregating these contributions. A commonly used kernel function is the normal kernel function.

Smoothing is controlled by a smoothing parameter, called the bandwidth parameter. For normal kernels, this value can be interpreted as the standard deviation for the normal distribution. A higher value leads to more smoothing, while a lower value leads to less smoothing.

Multiple methods are used in literature for selecting a bandwidth, ranging from rule-of-thumb estimators to cross-validation based estimators [1]. These methods generally give different results. In practice, one can try several methods and choose the smallest bandwidth value that provides appropriate smoothing for the intended type of analysis. We defined appropriate as not being able to visually identify individual kernels anymore.

*Temporal dimension*

In this study, the temporal dimension was defined as time of day (00:00-24:00 hours). To model this correctly, time must be circular in this formulation, like a clock. This was accomplished by choosing a von Mises kernel [2] to model the temporal dimension, which is an analogue to a normal distribution on a circle. Accordingly, time was represented as an angle θ in the circle, with time (00:00-24:00) mapped to values [-π, π].

Smoothing is controlled by the bandwidth parameter, also referred to as the concentration parameter in case of von Mises kernels. Here, a higher value leads to less smoothing, while a lower value leads to more smoothing, which is the inverse of the bandwidth parameter for normal kernels.

*Spatial relative risk*

The spatial relative risk estimate was defined as the natural logarithm of kernel density ratio of the non-survivors and survivors [3]. For this log spatial relative risk estimate ρ$\rho$, we found at any point in our study region:

- ρ$\rho$ > 0: relatively more non-survivors (i.e., higher relative risk of mortality)
- ρ < 0: relatively more survivors (i.e., lower relative risk of mortality)

Afterwards, areas of statistically significant high- or low-risk of mortality were calculated and were indicated by contour lines at significance level of 5% [3].

**Models**

*Model 1: Spatial distribution*

For the spatial analysis (X, Y coordinates), we defined a two-dimensional KDE model with normal kernels. The probability density that fell outside the municipal boundaries was redistributed using the boundary correction implementation of R package *sparr* [4]. Boundary corrected bandwidth methods based on the bootstrap estimate of the mean integrated squared error, unbiased least squares cross-validation, and likelihood cross-validation were tried and compared. The smallest bandwidth value that made individual kernels no longer identifiable was selected. The spatial analysis was performed in R (programming language).

*Model 2: Spatiotemporal distribution*

The spatiotemporal model extends Model 1 with a temporal dimension (angle $\theta$), based on the formulation of Brunsdon et al. [5]. Boundary correction was not used in the spatiotemporal model because of the addition of the temporal dimension. The temporal bandwidth was selected using the trigonometric moments method for Von Mises kernels for multimodal data [2]. We implemented the model and the temporal bandwidth selection method in Python (programming language).

*Model 3: Spatial relative risk*

The spatial bandwidth for the KDE of the non-survivors and survivors was selected using a jointly optimal common bandwidth [4]. Boundary correction was used in both spatial KDEs. The spatial relative risk analysis was performed in R (programming language).

**Bandwidth choice**

For the spatial model, a spatial bandwidth of 540.0 m was selected, obtained from the method that minimizes bootstrap estimate of the mean integrated squared error. For the spatiotemporal model, a spatial bandwidth of 635.5 m and a temporal bandwidth of 10.0 were selected. For the spatial relative risk analysis, a jointly optimal spatial bandwidth of 671.9 m was selected.

**References**

[1] Wang Q. Multivariate Kernel Smoothing and Its Applications. Journal of the American Statistical Association. 2020;115:486-.

[2] Taylor CC. Automatic bandwidth selection for circular density estimation. Computational Statistics & Data Analysis. 2008;52:3493-500.

[3] Hazelton ML, Davies TM. Inference Based on Kernel Estimates of the Relative Risk Function in Geographical Epidemiology. Biometrical Journal. 2009;51:98-109.

[4] Davies TM, Marshall JC, Hazelton ML. Tutorial on kernel estimation of continuous spatial and spatiotemporal relative risk. Statistics in Medicine. 2018;37:1191-221.

[5] Brunsdon C, Corcoran J, Higgs G. Visualising space and time in crime patterns: A comparison of methods. Computers, Environment and Urban Systems. 2007;31:52-75.
