# Supplementary material for: Where do we need to improve resuscitation? Spatial analysis of out-of-hospital cardiac arrest incidence and mortality
Source: Scand J Trauma Resusc Emerg Med. 2023 Oct 26;31:63. doi: 10.1186/s13049-023-01131-8 (PMC10605336; doi:10.1186/s13049-023-01131-8)

This supplemental document contains the results of the relative risk analysis based on the most recent 5 years of data and a table with yearly incidence and survival.

**Table 1**. Characteristics of out-of-hospital cardiac arrests in the high-, neutral-, and low-survival areas. (2012-2016)

|  |  |  |  |  |
| --- | --- | --- | --- | --- |
| **Variables** | **High-survival** | **Neutral-survival** | **Low-survival** | *P* |
| **n** | 183 | 945 | 257 |  |
| **Median age (IQR)** | 65 (56-73) | 66 (55-78) | 70 (61-81) | *<0.001* |
| **Male** | 78.7% (of 183) | 67.6% (of 945) | 61.9% (of 257) | *0.0026* |
| **Public** | 56.8% (of 183) | 26.7% (of 944) | 16.0% (of 257) | *<0.001* |
| **Witnessed** | 71.5% (of 179) | 69.0% (of 935) | 70.0% (of 253) | *0.8098* |
| **CPR before EMS** | 81.3% (of 176) | 77.6% (of 928) | 75.9% (of 249) | *0.2325* |
| **AED before EMS** | 67.8% (of 183) | 65.4% (of 944) | 62.3% (of 257) | *0.2765* |
| **Local AED** | 35.5% (of 124) | 13.6% (of 617) | 5.6% (of 160) | *<0.001* |
| **Shockable initial rhythm** | 51.7% (of 176) | 37.6% (of 889) | 28.6% (of 248) | *<0.001* |
| **30-day-survival** | 36.6% (of 183) | 21.7% (of 945) | 9.7% (of 257) | *<0.001* |
| **30-day-survival Utstein** | 68.8% (of 80) | 54.0% (of 291) | 29.5% (of 61) | *<0.001* |

Unknown or missing values were excluded for each variable separately and the numbers in parenthesis indicate the number of data points for each variable. CPR and AED rates reflect BLS and AED use before EMS arrival. Local AED refers to an on-site AED (instead of AEDs brought by first responders) and the corresponding value is defined only for the subgroup of cases where an AED is connected. 30-day survival is also given for the Utstein comparator group, i.e. witnessed arrests with shockable rhythm. All P-values except for age were obtained by a two proportion z-test comparing high and low-survival. P-value for age was obtained by a Kruskal-Wallis test. CPR: cardiopulmonary resuscitation, AED: automatic external defibrillator.

**Table 2**. Defibrillator connection time and time to first shock of out-of-hospital cardiac arrests in the high-, neutral-, and low-survival areas.

|  |  |  |  |  |  |
| --- | --- | --- | --- | --- | --- |
| **Variables** | | **High-survival** | **Neutral-survival** | **Low-survival** | *P* |
| **Defibrillator connection time** | **n** | 157 | 818 | 231 |  |
|  | **Mean** | 08:16 | 08:48 | 09:49 | 0.003 |
|  | **Median (IQR)** | 07:28  (05:45-10:31) | 08:03  (06:24-10:31) | 08:28  (07:01-11:11) |  |
| **Time to first shock** | **n** | 85 | 310 | 65 |  |
|  | **Mean** | 07:55 | 08:35 | 10:08 | 0.002 |
|  | **Median (IQR)** | 07:01 (05:23-09:16) | 08:06 (06:22-10:20) | 08:52  (07:10-11:24) |  |

Defibrillator connection time is retrieved from either a connected automatic external defibrillator or from the manual defibrillator of the Emergency Medical Service. Values exceeding 30 minutes were excluded from analysis in this table. P-values were obtained by a Kruskal-Wallis test.

**Table 4**. OR of the survival area variables, with and without adjustment for case characteristics.

| *Not adjusted for case characteristics* |  | OR | OR 95% CI | P |
| --- | --- | --- | --- | --- |
| High-survival |  | 2.31 | 1.63-3.27 | 2.72E-06 |
| Low-survival |  | 0.36 | 0.23-0.58 | 2.21E-05 |
|  |  |  |  |  |
| *Adjusted for case characteristics* |  | OR | OR 95% CI | P |
| High-survival |  | 1.58 | 0.99-2.52 | 0.05378 |
| Low-survival |  | 0.41 | 0.24-0.70 | 0.00112 |

**Table 5.** Yearly incidence and survival.

| **Year** | **n** | **Survived** | **% Survived** |
| --- | --- | --- | --- |
| 2006 | 267 | 51 | 19.1% |
| 2007 | 288 | 49 | 17.0% |
| 2008 | 280 | 61 | 21.8% |
| 2009 | 223 | 42 | 18.8% |
| 2010 | 238 | 41 | 17.2% |
| 2011 | 220 | 39 | 17.7% |
| 2012 | 221 | 47 | 21.3% |
| 2013 | 229 | 53 | 23.1% |
| 2014 | 296 | 68 | 23.0% |
| 2015 | 309 | 65 | 21.0% |
| 2016 | 330 | 64 | 19.4% |
|  | 2901 | 580 | 20.0% |

**Figure 1.** KDE
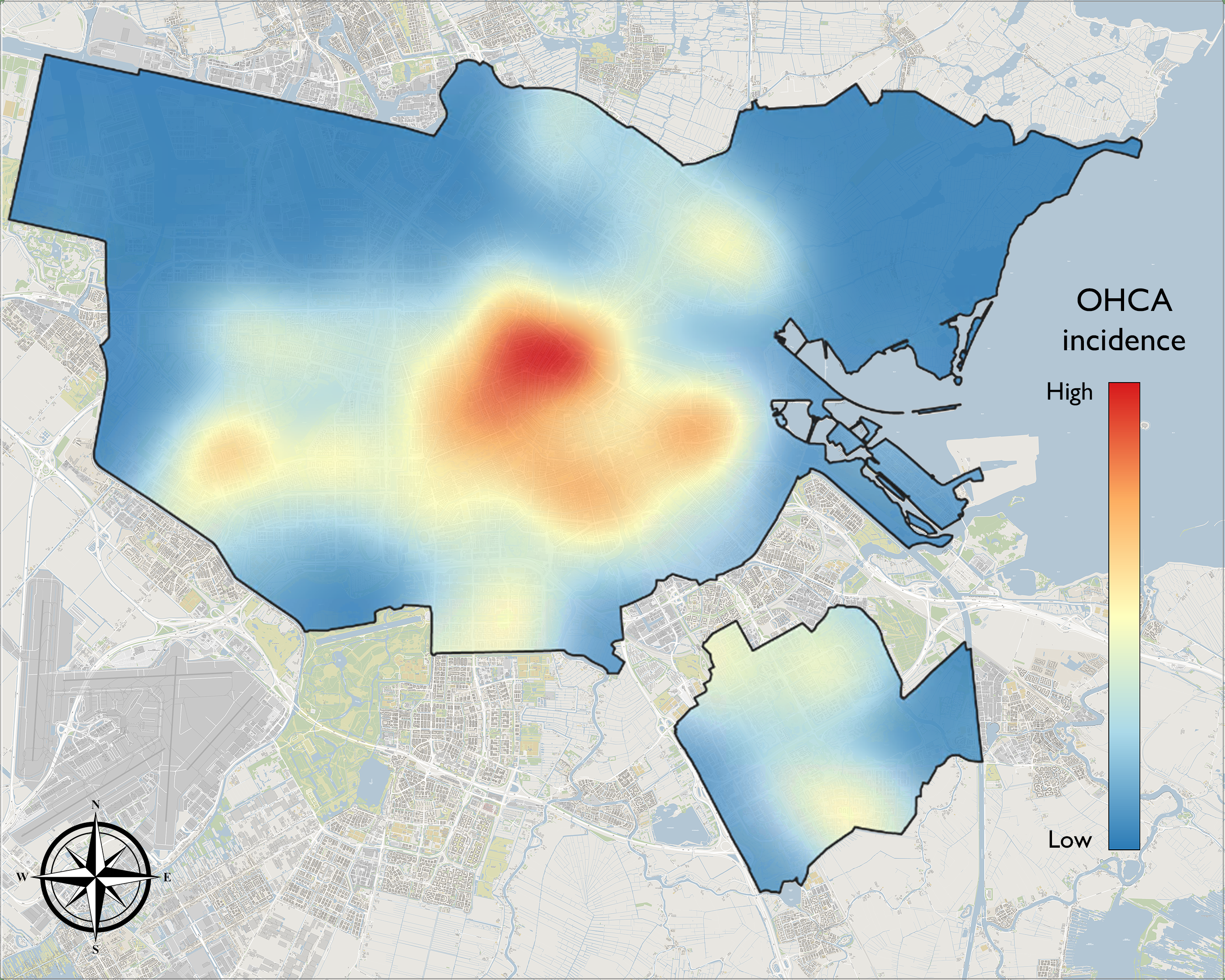


**Figure 2.** Relative risk. Contour lines of the last 5 years (bold) compared to contour lines of 11 years (transparent).


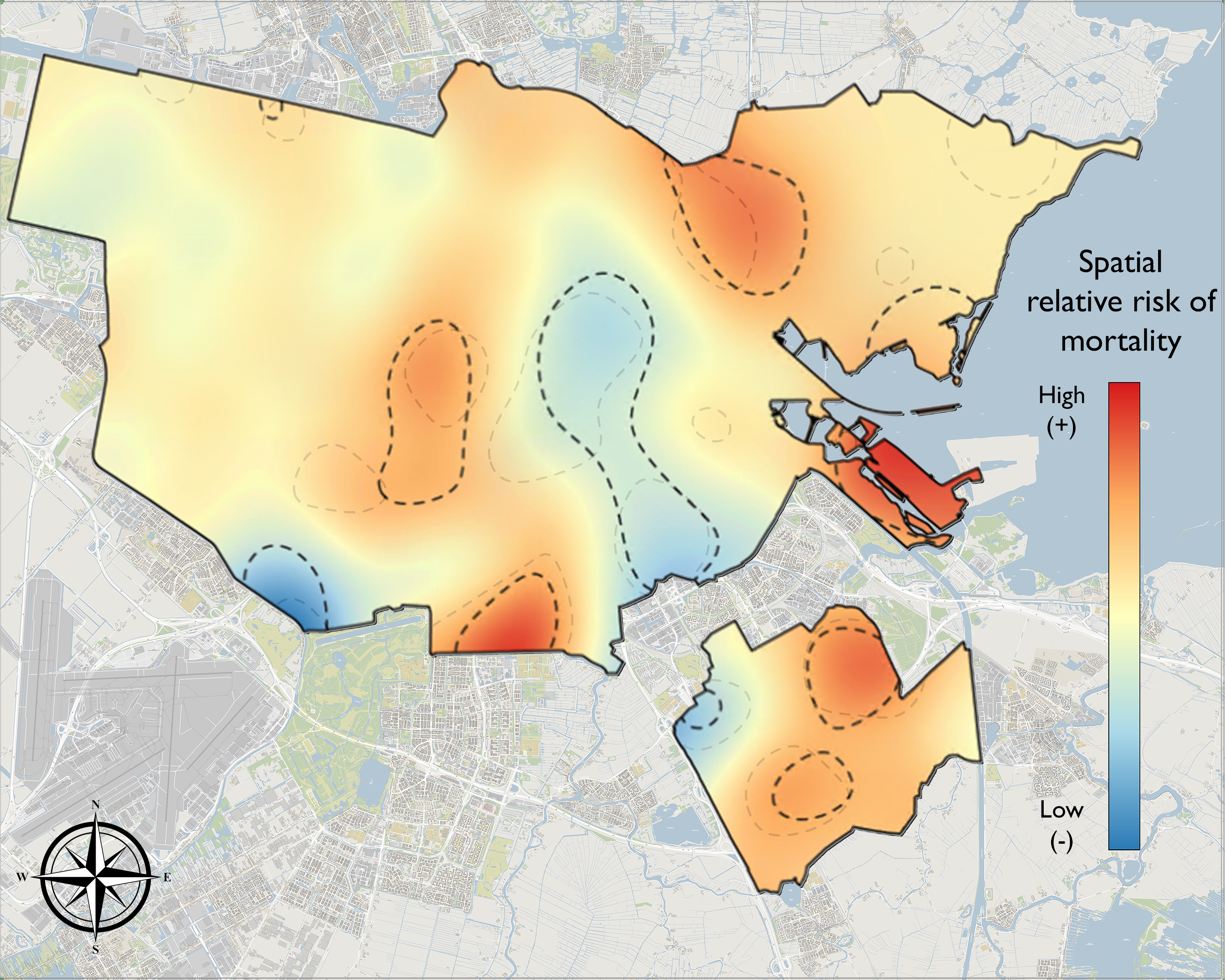

Supplement: Supplementary file 2 — Supplementary Material 2 [file 13049_2023_1131_MOESM2_ESM.docx]
